# Prevalence of *Caprine* brucellosis in Anhui province, China

**DOI:** 10.14202/vetworld.2019.558-564

**Published:** 2019-04-18

**Authors:** Sajid Ur Rahman, Lei Zhu, Li Cao, Yafei Zhang, Xiaoyan Chu, Shibin Feng, Yu Li, Jinjie Wu, Xichun Wang

**Affiliations:** Department of Clinical Veterinary Medicine, College of Animal Science and Technology, Anhui Agricultural University, 130 West Changjiang Road, Hefei 230036, China

**Keywords:** brucellosis, goats, polymerase chain reaction, prevalence, serological tests

## Abstract

**Background and Aim:**

Brucellosis is one of the most widespread zoonotic diseases globally. Studies indicated the existence of *Brucella* infection in goats in some province of China. Thus this study aimed to estimate the prevalence of brucellosis in goats of Anhui Province, China.

**Materials and Methods:**

Serum and milk samples obtained from goats in different regions of Anhui province were studied through rose Bengal plate test (RBPT), serum agglutination test (SAT), milk ring test (MRT), and polymerase chain reaction (PCR).

**Results:**

The investigation frequency of brucellosis using RBPT, SAT, MRT, and PCR methods was 3.9% (n=7), 4.45% (n=8), 11.67% (n=7), and 86.67% (n=156), respectively. The prevalence recorded for brucellosis in sex-wise animals as in females 5.55%, 6.67%, 11.67%, and 78.8% through above methods, while in males, it was 2.23% and 2.23% by RBPT and SAT. However, in age-wise animals, the results 6.36%, 7.27%, 11.67%, and 74.5% were perceived positive by RBPT, SAT, MRT, and PCR in adult females, respectively, but young males and females (up to 9 months) were considered free from brucellosis.

**Conclusion:**

These results show that prevalence of brucellosis was relatively higher in females than male’s goats and SAT was relatively specific and accurate as compared to RBPT and MRT, but for diagnosis of brucellosis, molecular method (PCR) is recommended.

## Introduction

Brucellosis is a zoonotic disease and an important public health problem in different parts of the world, particularly in the Middle East region [1]. More than 500,000 human brucellosis cases are reported each year globally, but the number of undetected patients is believed to be significantly higher [2]. The traditional epidemiology of this zoonotic disease has altered dramatically over the past two eras, related to major political and socioeconomic events. Thus, while the prevalence remains high in the Middle East and North African countries, it has been greatly reduced in Latin America and South European countries. *Caprine* is a small ruminant, raised for different purposes such as meat, milk, and hair production, mainly in dry, humid, and mountainous temperate regions of numerous countries [3]. In the year 2009, a total of 35,816 cases of brucellosis were found in different parts of China [4]. Previous researches have stated serological signs of brucellosis in China between humans who had close contact with domestic animals such as goats [5,6]. However, rare evidence is available about the incidence of *Brucella* infection in goats in Anhui province.

Brucellosis is a foremost evolving zoonosis caused by small, non-motile Gram-negative and intracellular coccobacilli belong to the genus *Brucella* [7-9]. Brucellosis is caused by four key etiological mediators such as *Brucella* *abortus* (BA), *Brucella* *melitensis* (BM), *Brucella* *canis*, and *Brucella* *suis*, affecting different animals and humans. Numerous intracellular pathogens, such as BA, show a biphasic infection course starting with a non-proliferative stage of unclear nature [10]. Abortion, infertility, sterility, and drop in milk production are the problems that caused severe economic losses due to this disease [11]. The transmission of *Brucella* organisms between animals is usually by contact with the placenta, fetus, fetal fluids, and vaginal discharges from a diseased animal. Entry into the body happens by consumption and through the mucous membranes, damaged skin, and possibly intact skin [12]. Numerous species of *Brucella* distress in different animals. Humans are particularly infected through contacted directly with animals or animal’s products previously contaminated with *Brucella* species or such kind of bacteria. In humans, brucellosis triggered a different kind of diseases related to flu as well as comprise fever, sweats, headache, back pain, and physical weakness. There should be severe infections in the central nervous system, including in the lining of the heart. Long-lasting indications such as fatigue, recurrent fever, and joint pain also occur due to Brucellosis. The symptoms of brucellosis in humans comprise malaise, fever (39-40°C), faintness, pain, backache, anorexia as well as loss in weight. A kind of fever called undulant fever can last for weeks to years when there is *Brucella* in the body [13].

The accurate diagnosis of brucellosis is still a challenge for many researchers as it entirely based on serological recognition of BA and BM. There are different ways to diagnosed *Brucella* species such as microscopic assessment of stained smears from aborted tissue or cultured material, phage typing, and immunofluorescence staining, but polymerase chain reaction (PCR) is usually used [14]. Various serological tests such as serum agglutination test (SAT), plate agglutination, Rivanol agglutination test, complement fixation test, *Brucella* ring test, and enzyme-linked immunosorbent assay are used to investigated Brucellosis. Serological procedures are the pillars for analysis and mass testing programs [15]. Among different tests, the utmost successful tests for BA and BM are based on the uncovering of antibodies to lipopolysaccharide antigen of smooth *Brucella* strains [16,17]. The clinical history of brucellosis is not pathognomonic, and the clinical history of the patient is of supreme importance in diagnosis. Undisputable diagnosis of *Brucella* infections can be made only by the separation and identification of *Brucella*, but in conditions where the bacteriological investigation is not practicable, the finding is accepted using serological methods [18].

The presence of *Brucella* infection occurs in individuals due to a direct interface with diseased *Caprine* herds, manure, milk as well as its by-products. However, no information in this favor was available that makes the goat population a susceptible health risk for humans. The present study was, therefore, intended to estimate the prevalence of brucellosis in goats of Anhui Province China.

## Materials and Methods

### Ethical approval

The experimental protocol was approved by Anhui Agricultural University Animal Care and Institutional Animal Ethical Committee (ZXD-C2018520) (Hefei, China).

### Study area

Anhui province is situated on the east side of China. It is the 8^th^ most crowded and 22^nd^ big Chinese province based on area, in the all 34 Chinese provincial areas. Its capital is Hefei which is the second largest city in Anhui province. Anhui comprises a huge area of grasslands, and sheep and goats are either foraged distinctly or collectively under a common foraging system. As with landscape, Anhui varies in climate from south to north. The north is more moderate and has more clear-cut periods. In January, temperatures average at around −1-2°C and 0-3°C. In July, temperature averages 27°C or more. The study location comprised seven of all 16 districts of Anhui. The study was conducted between March and June 2018.

### Collection of blood and milk samples

A total of 240 samples, 180 bloods, 90 from each male and female goat and 60 milk samples were also collected from the same animals (females) from seven different regions of Anhui province China, for the current research. Blood samples of 10 ml were obtained using a sterile vacutainer tube from the jugular veins of the goats and were divided into two tubes, the first containing the anticoagulant EDTA, and the other without anticoagulant for PCR assay and serum separation. The whole blood samples collected for the PCR assay were stored at −20°C till analysis. The serum samples were aliquoted and stored at −80°C until analysis.

### DNA preparation and PCR assay

**Figure-1:**
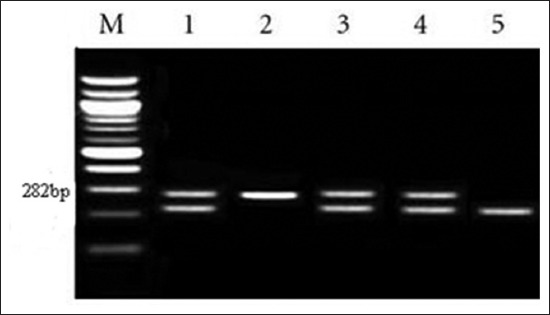
Agarose gel electrophoresis of polymerase chain reaction was performed for detection of Brucella in goat’s samples. The figure shows the uncut 282 bp DNA. M: molecular 100 size ladder (in base pairs), the 1^st^ lane, 3^rd^ lane, 4^th^ lane = Positive control, 2^nd^ = Positive specimens, 5^th^ lane= Negative control.

Highly pure PCR template preparation kit™ (Roche Life Science) was used to purify DNA according to manufacturer instruction. For the assay, about 200 µl of whole blood was used. DNA concentration was determined spectrophotometrically using NanoDrop ND-1000 ultraviolet (UV) (Nano-Drop technology, Wilmington, USA). Specific PCR assays for BA and BM were performed in single runs [19]. According to the Gene Bank sequence, the primer sequence of the target genes (i.e., BA=Forward primer 5′GCGGCTTTTCTATCACGGTATTCR3′ Reverse Primer 5′–3′CATGCGCTATGATCTGGTTACG and BM=Forward Primer5′–3′AACAAGCGGCACCCC TAAAA, Reverse Primer5′3′CATGCGCTATGATC TGGTTACG), these primers were used before by Wareth *et al*. [17]. Primers were utilized in a 0.75 µl reaction containing 12.5 µl of Emerald Amp Max PCR Master Mix (TakaRa, Dalian, China), 0.25 µl probes and 4.5 µl of DNA template which was filled with 4.5 µl of water. Real-time quantitative PCR with decontamination at 50°C for 2 min single cycle and denaturation at 95°C for 10 min 50 cycles were performed, annealing for 1 min at 50°C for primers. The last cycle comprised incubation of the sample at 72°C for 10 min and was kept at 4°C for limitless time. About 7 ml of the augmented product were examined by electrophoresis in ethidium bromide stained 1.5% agarose gel. After this, the amplified product was imagined underneath UV light and then was imaged using Alphalmager (Alpha Innotech). All the samples were measured in duplicate. The values <40 cycles for threshold cycle were considered as positive (Figure-1).

### Rose Bengal plate test (RBPT)

The obtained Rose Bengal stained comprising BA cells (strain 99) (ID. vet innovative diagnostics, France) were deferred in the buffer at pH 3.6. To bring the sample into their normal state, all the antigen and samples of serum were kept at room temperature. The procedure was performed as described by Shahaza *et al*. [20]. First of all, 0.03 ml serum was put on a transparent glass slide for BA, while the same procedure was applied on another slide for BM. Likewise, a drop of negative and positive control serum was located distinctly on the slide. The ampoule was shaken slightly to make an undeviating suspension, and then 0.03 ml amount of antigen suspension was taken from the ampoule and sited on slide close to the drops of serum. First, the slide was macroscopically observed using telescopic glass for cohesion under a dressed source of light in contradiction of a dim background arena. The positive interface among antigen and serum, as well as granules presence with dissimilar strength, identified the level of antibodies in the serum of the animal diseased with precise species of bacterial organism.

### Milk ring test (MRT)

This method was previously described by Islam *et al*. and Khan *et al*. [21,22]. For identification of brucellosis through MRT, hematoxylin with BA strain 99 antigen was used. After fitting the test tube in the test tube rack, a sample of 1 ml of milk was dispersed into each test tube. There were another two test tubes which were used as a control antigen, one of which comprises *Brucella* positive milk sample (control). A 0.05 ml volume of the MRT stained antigen (hematoxylin) was added to each tube. The racks were shaken moderately, and the antigen and test milk sample were assorted carefully. After this, these samples were permitted not to disturbed for around 2 min and kept in an incubator at 37°C for 1 h, and the result was recorded after 1 h.

### SAT

The SAT was performed rendering to the process described previously by Alton *et al*. [23]. Antigen was prepared and standardized according to the procedure given by Morganville, diagnostic laboratory, USA. Ten test tubes of 12 mm×75 mm were placed in an appropriate test tube rack. During the starting of the procedure, 1.9 ml of 0.9% of sodium chloride solution were added to the first test tube, and then, 1.0 ml of physiological solution and test serum were added to the remaining tubes, mixed thoroughly and 1.0 ml of the diluted serum were transferred from the 1^st^ to 2^nd^ and from 2^nd^ to 3^rd^ tube. The same process was repeated up to 10 tubes. A diluted serum of 1 ml was discarded from tube 10 but left 1 ml. All the 10 tubes contained 1.0 ml each of serial two-fold dilutions of 1:2-1:10240, without tube number 1 which was considered as a 1:20 dilution. A test tube contained 1.0 ml; physiological solution was placed at the end of the row and labeled as saline control. A series of dilutions, as carried out for the positive control sera and negative as well. The antigen suspension was mixed well, shaken and then a drop of antigen was added to each tube. The antigen and serum were mixed well by shaking the rack and then placed in a water bath and incubated at 37°C for 48 h. After incubation care was taken to not disturb the agglutination. All tubes were examined using an indirect source of light against a dark background.

### Statistical analysis

To determine the diagnosis of Brucellosis, goats were considered positive if they showed a positive reaction in at least one serological test or PCR. The collected data were analyzed using the percentage formula: Number of positive goat’s ÷ total number of goat’s×100. For PCR, data analysis was performed using statistical software (SPSS for Windows, Version 17.01, SPSS Inc., Chicago, USA).

## Results

**Figure-2:**
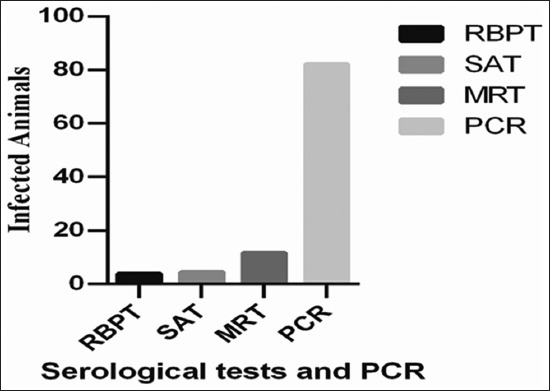
Overall brucellosis examined by different serological tests in 180 samples. The data expressed as percentage (100%). For abbreviation please see footnote of Table-1.

The total positive brucellosis cases in 180 samples recorded are 7 positive (3.9%), 8 positive (4.45%), and 156 positive (86.67%), by RBPT, SAT and PCR, but through MRT we examined 60 samples out of which 7 showed positive cases (11.67%) (Figure-2).

The data regarding the diagnosis and prevalence of brucellosis in male and female goats are presented in Table-1. 90 male and 90 female goats were examined by RBPT and SAT and 60 milk samples from female goats by MRT. The diagnosis of brucellosis in female *Caprine* was recorded as 5.55%, 6.67%, 11.67%, and 78.8% by RBPT, SAT, MRT, and PCR, respectively, while, in males, it was detected as 2.23% and 2.23% by both RBPT and SAT. PCR was presented negative results in all samples. The collected milk samples investigated for the prevalence of brucellosis, MRT diagnosed 11.67% brucellosis in females (Table-1).

**Table-1:** Sex-wise diagnosis of brucellosis in goats determined by various techniques used during the experiments.

Techniques used	Females goats			Male goats		
	
	TNGE	NPG	PPG	TNGE	NPG	PPG
RBPT	90	05	5.55	90	02	2.23
SAT	90	06	6.67	90	02	2.23
MRT	60	07	11.67	-	-	-
PCR	90	71	78.8	-	-	-

n=240, TNGE=Total No. of goats examined, NPG=No. of positive goats, PPG=Percent of positive goat, RBPT=Rose Bengal plate test, SAT=Serum agglutination test, MRT=Milk ring test, PCR=Polymerase chain reaction

### Brucellosis in different ages of goats

The data in Table-2 showed that positive cases of brucellosis in adult goats (above 9 months-1.5 years) were observed as 6.36%, 7.27%, 11.67%, and 74.5% by RBPT, SAT, MRT, and PCR, respectively, while all young goats (below 9 months) were found to be negative (Table-2).

**Table-2:** Age-wise diagnosis of brucellosis in goats determined by various tests used during the current investigation.

Techniques used	Adult goats			Young goats		
	
	TNYGE	NPG	PPG	TNYGE	NPG	PPG
RBPT	110	7	6.36	30	00	0
SAT	110	8	7.27	30	00	00
MRT	60	7	11.67	-	-	-
PCR	110	82	74.5	-	-	-

TNYGE=Total number of young goats examined, NPG=Number of positive goats, PPG=Percent of positive goat, RBPT=Rose Bengal plate test, SAT=Serum agglutination test, MRT=Milk ring test, PCR=Polymerase chain reaction

A total of 55 adult female goats (above 2 years) were analyzed through RBPT, SAT, and MRT which detected 5.45%, 7.27%, and 9.0% brucellosis while remaining were recorded as negative or free from disease presented in Table-3. During the survey, 20 young females (up to 5 months) were also examined through RBPT and SAT but failed to detect any positive sign and considered to be negative. MRT was not carried out because animals were very young and unable to get any milk sample (Table-3).

**Table-3:** Diagnosis of brucellosis in different categories of goats determined by various techniques used during the present investigation.

Techniques used	AFG			YFG		
	
	AFG	PG	PPG (%)	YFG	PG	PPG (%)
RBPT	55	03	5.45	20	00	00
SAT	55	04	7.27	20	00	00
MRT	55	05	9.0	-	-	-

**Techniques used**	**AMG**			**YMG**		
	
	**AMG**	**PG**	**PPG**	**YMG**	**PG**	**PPG**

RBPT	55	02	3.63	20	00	00
SAT	55	02	3.63	20	00	00
MRT	-	-	-	-	-	-

AFG=Adult female goats, PG=Positive goats, PPG=Percentage of positive goats, YFG=Young female goats, AMG=Adult male goats, YMG=Young male goats, RBPT=Rose Bengal plate test, SAT=Serum agglutination test, MRT=Milk ring test

### The prevalence of Brucella species in goats

**Figure-3:**
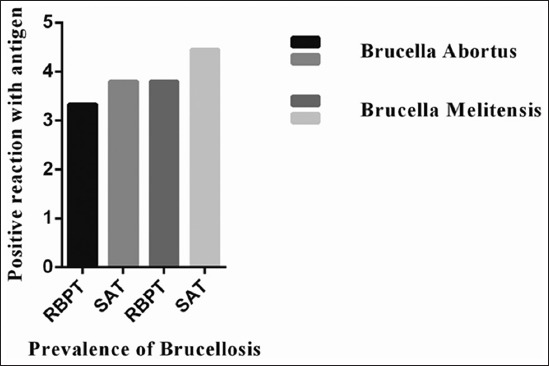
Animals reacted with *Brucella melitensis* antigen showed a positive reactor of 3.8% through rose Bengal plate test (RBPT) and 4.45% by serum agglutination test (SAT) as well as *Brucella abortus* indicated 3.33% positive reactor by RBPT and 3.8% SAT. The data expressed as percentage (100%).

The result in Figure-3 indicated the prevalence of *Brucella* species recognized through antibodies in the serum of goats infected with different species. The species and their prevalence in the serum of goats were diagnosed by RBPT in which 180 goat serum samples were tested for BA (IS711) and BM (IS711) antibodies. BA antigen interacted with six sera contained the antibodies of BA species. Serologically, the occurrence of BA and melitensis was recorded as 3.33 and 3.8%, respectively. Furthermore, it was also observed that the sera, which interrelated to BA antigen, were also interacted to melitensis and concluded to be cross reactivation which is the evidence in both organisms. This might be the presence of antibodies of the species which previously infected the goats, or this might be the result of cross-reaction properties present in two closely related species that misled the result in two different species. It was evident that only one serum sample which interacted strongly with melitensis and declared as positive for melitensis. Similar findings were also evidence for SAT where both BA and melitensis showed a close level of incidence in the sera of goats. On the other hand, only six serum samples which comprise melitensis antibodies that interacted positively with the specific antigen of melitensis and showed a positive reactor of 3.8% by RBPT and 4.45% SAT are presented in Figure-3.

## Discussion

Brucellosis is a transmissible ailment of livestock with substantial economic impact. Brucellosis is caused by numerous bacteria of the *Brucella* family, which incline to contaminate a precise animal species or humans [24]. The causal mediator of a zoonotic disease called brucellosis is *Brucella* that affects millions of peoples globally and shows a different kind of symptoms when transmitted to human or animal’s body due to sRNAs that are involved in virulence and stress adaptation of *Brucella* [25]. An investigation by serological or other tests, and on milk like the MRT, can be used for showing and plays a significant role in campaigns to eradicate this zoonotic disease. Separate animal investigation both for trade and for disease eradication purposes is practiced [24]. During the present investigation, brucellosis recorded by RBPT, SAT, MRT, and PCR was 3.9%, 4.45%, 11.67%, and 86.2%, respectively. Saadat *et al*. and Bale *et al*. [26-28] diagnosed similar percentage of brucellosis in goats, but in the present study, MRT detects higher result (11.67%) as compared to RBPT and SAT both diagnosed 3.9 and 4.45%. In the current investigation, we examined 90 male and female goats through RBPT, SAT, and PCR while 60 milk samples were inspected by another test called MRT. In females, its diagnosis was relatively recorded higher of about 5.55%, 6.67%, 11.67%, and 78.2% through RBPT, SAT, MRT, and PCR assay, respectively [19]. Our research finds higher prevalence in females as compared to males. The similar percentage of brucellosis was also observed by Nagati *et al*. [29] in different sexes of *Caprine*. Numerous investigators noted brucellosis moderately higher percentage and rather lower than our study. Therefore, our findings could not be associated and defendable for male and female brucellosis prevalence under different management and husbandry circumstances where the animals are raised and reserved for the basis of profits. Our study could also be helpful in planning and eradication of brucellosis in goats in the area. Larger goat herds have a higher probability for contact between the animals, and similar epidemiological reviews outcomes had been formerly stated in different animals [30]. Our study observed 110 adult goats (age more than 9 months) through RBPT, SAT, and PCR as well as 60 mature female goats by MRT while 30 young goats (around 9 months of age) by RBPT and SAT. The positive brucellosis recorded in adult goats was 6.36%, 7.27%, 11.67%, and 74.7% by RBPT, SAT, MRT, and PCR individually. All the young goats were found free from brucellosis, and we did not find any symptom of this zoonotic disease (Table-2). It is suggested that for lactating animals SAT and MRT could be applied for investigation of brucellosis. A total of 55 adults female goats were examined through by RBPT, SAT, and MRT to diagnosed brucellosis in different categories of goats [31]. The brucellosis which is diagnosed 5.45%, 7.27%, and 9.09% adults’ female goats, respectively, while remaining goats were considered negative or free from any brucellosis (Table-3). During present research, 20 young females were also examined through RBPT and SAT; both techniques were failed to detect any brucellosis and considered to be negative. The animals are too young and unable to get any milk sample, so MRT was not carried out. During these observations, young male goats were also examined by RBPT and SAT in which all young male goats were found free from brucellosis (Table-3). One should conclude that the prevalence of brucellosis in different categories of goats depends on the husbandry conditions of the animals. Furthermore, the geographical condition where the samples were taken varies with their environmental conditions which might suit the species to cause infection in different categories of animals. Second, the young animals might have their strong immunity which provides support to resist against *Brucella* agents to cause infections as compared to adults. The diagnostic accuracy of the MRT was compared with the SAT and RBPT because it can detect more positive cases as compared to both tests. Based on the study of Akhtar *et al*. [32], the increasing number of positive cases in MRT may be due to many factors such as antigen purity, storage temperature contamination, vaccines status of animals, and infection with other phylogenetically related bacteria. In general, 6.9% of the goats were detected as positive for brucellosis confirmed by SAT [33]. All infected goats showed more than or 1:40 (I.U) antibody titer in their serum. Figure-2 shows that BA antigen interacted with six serum samples restricted the BA antibodies with a positive reactor of 3.33% by RBPT and SAT. These results are consistent with findings of Hashemi *et al*. [34], who found that culture of blood and the clinical specimens are useful for definitive diagnosis even in patients with low titers of antibodies. During the present study, only six serum samples which comprise melitensis antibodies that interacted positively with the specific antigen of melitensis and show a positive reactor of 3.8% by RBPT and 4.45% SAT. Serologically, the incidence of BA and melitensis with a positive reactor of 3.33% by RBPT and 3.33% by SAT was recorded in goats, respectively [35].

To diagnose brucellosis needs separation of the bacteria or endorsement through serological methods. However, culture specimen sensitivity is frequently low, depending on the severity of the disease. The development of the PCR has presented a novel measurement for the diagnosis of many microorganisms and is possible in just a little time as compared to other serological tests such as RBPT, SAT, and MRT.

## Conclusion

The present study concluded that the prevalence and diagnosis of brucellosis were somewhat higher and increased in female as compared to males. There was no brucellosis diagnosed in young goats. It was also concluded from the present research that SAT is specific and quite more accurate and cheap, but for the diagnosis of brucellosis, PCR is recommended. Further studies should be needed to know why the prevalence rate is higher in female goats.

## Authors’ Contributions

SUR and XW conceived and designed the research. LZ, LC, YZ, and XC conducted the sample collection. SF, YL, and JW reviewed the manuscript. SUR and XW carried out the data analysis and writing of the manuscript. All authors read and approved the final manuscript.
